# Prognostic impact of tumour-specific HMG-CoA reductase expression in primary breast cancer

**DOI:** 10.1186/bcr2146

**Published:** 2008-09-22

**Authors:** Signe Borgquist, Annika Jögi, Fredrik Pontén, Lisa Rydén, Donal J Brennan, Karin Jirström

**Affiliations:** 1 Center for Molecular Pathology, Department of Laboratory Medicine, Malmö University Hospital, Lund University, SE-205 02 Malmö, Sweden; 2 Division of Oncology, Department of Clinical Sciences, Lund University Hospital, SE-221 85 Lund, Sweden; 3 CREATE Health Center for Translational Cancer Research, Lund University, SE-221 84 Lund, Sweden; 4 Department of Genetics and Pathology, Rudbeck Laboratory, Uppsala University, Uppsala, SE-751 85 Sweden; 5 Division of Surgery, Department of Clinical Sciences, Lund University Hospital, SE-221 85 Lund, Sweden; 6 UCD School of Biomolecular and Biomedical Science, UCD Conway Institute, University College Dublin, Belfield, Dublin 4, Ireland

## Abstract

**Introduction:**

We have previously reported that tumour-specific expression of the rate-limiting enzyme, 3-hydroxy-3-methylglutharyl-coenzyme A reductase (HMG-CoAR), in the mevalonate pathway is associated with more favourable tumour parameters in breast cancer. In the present study, we examined the prognostic value of HMG-CoAR expression in a large cohort of primary breast cancer patients with long-term follow up.

**Methods:**

The expression of HMG-CoAR was assessed by immunohistochemistry on tissue microarrays with tumour specimens from 498 consecutive cases of breast cancer with a median follow-up of 128 months. Kaplan Meier analysis and Cox proportional hazards modelling were used to estimate the rate of recurrence-free survival (RFS) and breast cancer specific survival (BCSS).

**Results:**

In line with our previous findings, tumour-specific HMG-CoAR expression was associated with low grade (p < 0.001), small size (p = 0.007), oestrogen receptor (ER) positive (p = 0.01), low Ki-67 (p = 0.02) tumours. Patients with tumours expressing HMG-CoAR had a significantly prolonged RFS, even when adjusted for established prognostic factors (relative risk [RR] = 0.60, 95% confidence interval [CI] 0.40 to 0.92; p = 0.02). In ER-negative tumours, however, there was a trend, that was not significantly significant, towards a shorter RFS in HMG-CoAR expressing tumours.

**Conclusions:**

HMG-CoAR expression is an independent predictor of a prolonged RFS in primary breast cancer. This may, however, not be true for ER-negative tumours. Further studies are needed to shed light on the value of HMG-CoAR expression as a surrogate marker of response to statin treatment, especially with respect to hormone receptor status.

## Introduction

The enzymatic activity of 3-hydroxy-3methylglutharyl-coenzyme A reductase (HMG-CoAR) is elevated in cancer cells [1]. HMG-CoAR acts as a rate-limiting enzyme in the mevalonatepathway, in which the main product is cholesterol. However, the pathway also produces a number of non-sterol isoprenoid side products, which have been shown to be important regulators of several oncogenic properties including angiogenesis, proliferation and migration [2,3]. Thus, increased levels of tumour-specific HMG-CoAR might reflect an increased demand of isoprenoids to maintain growth advantages within the cancer cell [1].

HMG-CoAR inhibitors, also known as statins, commonly used in the treatment of hypercholesterolaemia, have demonstrated anti-neoplastic effects *in vitro *[4-6]. Both the isoprenoid-mediated anti-tumoural effects and the cholesterol-lowering effects of statins have been suggested to lower the incidence of cancer among statin users [7]. Epidemiological studies have not been able to agree on an association between statin use and overall breast cancer risk [8,9]; however, a lower incidence of oestrogen receptor (ER) negative tumours has been reported among statin users [10]. Furthermore, an inverse relationship between statin use after diagnosis and breast cancer recurrence has been reported [11].

In a recently published study [12], we investigated the tumour-specific expression of HMG-CoAR by immunohistochemistry in 511 cases of incident breast cancer within the population-based prospective cohort of the Malmö Diet and Cancer Study (MDCS) [13]. This study demonstrated that HMG-CoAR was expressed at various intensities in 82% of the tumours and increased levels of HMG-CoAR protein expression were associated with favourable tumour characteristics such as a smaller tumour size, low histological grade and ER positivity. However, due to a small number of breast-cancer related events in the MDCS, it was not possible to perform survival analyses in relation to expression of the tumour-specific, HMG-CoAR protein. In the present study we therefore aimed to analyse HMG-CoAR protein expression by immunohistochemistry in a consecutive cohort of 498 patients with invasive breast cancer with long-term follow-up. The aim of this study was to examine the relationship between HMG-CoAR expression and disease outcome as well as established clinicopathological parameters.

## Materials and methods

### Patients

This study included 498 patients with primary invasive breast cancer treated and diagnosed at the Malmö University Hospital between 1 January 1988 and 31 December 1992. The cases belonged to an original cohort of 512 patients [14]. The median age at diagnosis was 65 years (range 27 to 96 years) and median follow-up time to first breast cancer event was 128 months (range 0 to 207 months). Information regarding the date of death was obtained from the regional cause-of-death registries for all patients. Complete treatment data were available for 379 (76%) patients, 160 of whom had received adjuvant tamoxifen. Information on adjuvant systemic chemotherapy was available for 382 patients, of which only 23 patients had received treatment. Two hundred patients received no adjuvant systemic treatment. Ethical permission was obtained from the Local Ethics Committee at Lund University (Dnr 613/02), whereby informed consent was deemed not to be required, but opting out was an option.

### Tissue microarray construction

For the present study, new tissue microarrays (TMAs) were constructed as described previously [15]. In brief, two 1.0 mm cores were taken from areas representative of invasive cancer and mounted in a recipient block using a manual arraying device (MTA-1, Beecher Inc, WI, USA).

### Immunohistochemistry

As described previously[16], sections 4 μm in diameter were dried, deparaffinised, rehydrated and treated in a microwave for two rounds of five minutes in citrate buffer before being stained in a Techmate 500 (DAKO, Copenhagen, Denmark) with a polyclonal anti-HMG-CoAR antibody (Catalog # 07-457, Upstate) diluted 1:250.

For all other antibodies, heat-mediated antigen retrieval was performed using microwave treatment for two rounds of five minutes in a citrate buffer before being processed either in the Ventana Benchmark system (Ventana Medical Systems Inc, AZ) using pre-diluted antibodies to ER (Anti-ER, clone 6F11), progesterone receptor (PR; Anti-PgR, clone 16) and human epidermal growth factor receptor 2 (HER2; Pathway CB-USA, 760-2694) or in the Dako Techmate 500 system (Dako, Glostrup, Denmark) for Ki-67 (1:200, M7240; Dako, Glostrup, Denmark).

Cytoplasmic staining of HMG-CoAR was assessed both as the fraction of positive cells (0 to 1%, 2 to 10%, 11 to 50% and 51 to 100%) and the staining intensity in the cytoplasm (negative = 0, weak = 1, moderate = 2, strong = 3). ER, PR, HER2 and Ki-67 were assessed as previously described [14]. ER and PR negativity was defined as less than 10% positively staining nuclei, according to current clinical guidelines in Sweden.

### Statistical analysis

Differences in distribution of clinical data and tumour characteristics between HMG-CoAR-negative and HMG-CoAR-positive tumours were evaluated using the chi-squared test. The Kaplan-Meier analysis and the log rank test were used to illustrate differences between recurrence free survival (RFS) and breast cancer-specific survival (BCSS) according to HMG-CoAR expression. Cox regression proportional hazards models were used to estimate the impact of HMG-CoAR expression on RFS and BCSS in both univariate and multivariate analysis, adjusted for tumour size, ER, HER2, lymph node status and Nottingham histological grade (NHG) of the entire cohort. Repeated analyses were performed on ER-positive and ER-negative patients separately. The null hypothesis of prognostic effects of HMG-CoAR status in ER-positive and ER-negative patients were evaluated using a Cox model with a term for the interaction between ER status and HMG-CoAR status (ER status [+/-] X HMG-CoAR status [+/-]) also included adjustment for established prognostic factors. All calculations were performed using SPSS version 15.0 (SPSS Inc, Chicago, IL). All statistical tests were two-sided and a p < 0.05 was considered statistically significant.

## Results

### Immunohistochemical HMG-CoAR expression in invasive breast cancer

**Figure 1:**
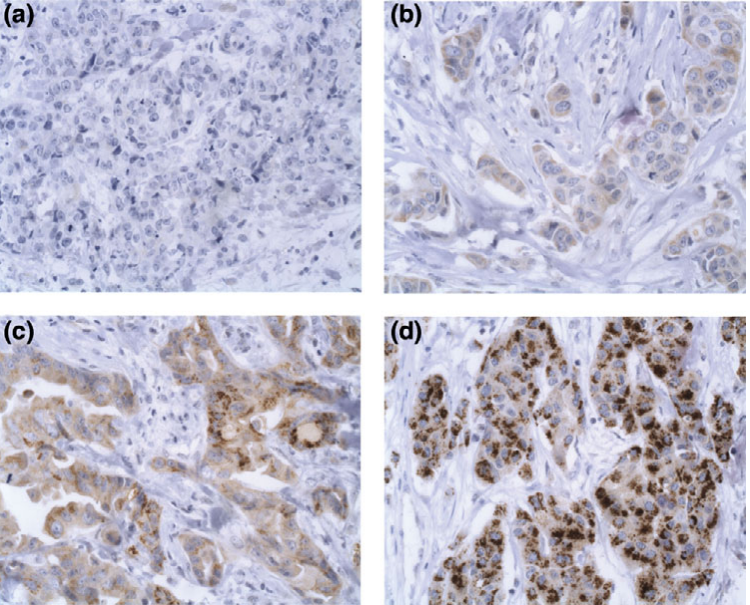
**Immunohistochemical images of HMG-CoA reductase staining**. Images (×400) representing tumours with (a) negative, (b) weak, (c) moderate and (d) strong immunohistochemical staining intensity for HMG-CoA reductase.

The specificity of the anti HMG-CoAR antibody was confirmed in a previous study [16]. In the current study, HMG-CoAR expression was evaluated in 444 cases. On the TMA (n = 54) missed tumour cores were either lost during immunohistochemistry processing or did not contain invasive cancer. When present, HMG-CoAR was generally expressed in the majority of tumour cells (>50%) and, therefore, only the staining intensity was accounted for in the statistical analyses. One hundred and eleven (25%) tumours lacked HMG-CoAR expression (fraction of positive cells <1%), 204 (45.9%) demonstrated weak expression, 117 (26.4%) had moderate expression and 12 (2.7%) had a strong signal. Figure 1 shows examples of tumours with different staining intensities, from negative to strong staining. In some cases, the staining was accentuated towards the membrane and a few cases displayed a coarse, granular cytoplasmic staining. Different subcellular localisations were, however, not accounted for in the statistical analyses.

### Correlation between HMG-CoAR and relevant clinicopathological parameters

In order to confirm and validate our previous findings [16], HMG-CoAR protein expression data were dichotomised into absent staining versus any staining. As demonstrated in table 1, and in line with previous findings, HMG-CoAR expression was associated with a smaller tumour size (p = 0.007), low histological grade (p < 0.001), ER positivity (p = 0.01) and low proliferation (Ki67; p = 0.02). No association was evident between HMG-CoAR expression and lymph node status, patient age or PR status. We used a 10% cut-off to determine hormone receptor (HR) status; however, comparison of HMG-CoAR expression with more defined categories of HR positivity, for example 0 to 10%, 11 to 50%, 51 to 75% and more than 75%, did not change these associations considerably, as did neither ER (p = 0.04) or PR (0.88). The correlations shown in table 1 refer to absent versus present HMG-CoAR expression, corresponding to the dichotomised variable used for survival analyses, but similar associations were seen between HMG-CoAR expression and relevant clinicopathological parameters when the former was categorised as 0 to 4, as in the previous study [12] (data not shown).

**Table 1:** Correlation betweeen HMG-CoAR expression and clinicopathological parameters.

**HMG-CoAR intensity**	**0**	**1 to 3**	
n (%)	111 (25.0)	333 (75.0)	p value
**Age (years)**			
Median	65	64	
Range	27 to 89	34 to 96	0.84
≤50	23 (20.7)	51 (15.5)	
>50	88 (79.3)	279 (84.5)	0.20
**Tumour size (mm)**			
Median	20	15	
Range	1 to 100	1 to 100	<0.001
≤20 mm	57 (51.4)	217 (65.8)	
>20 mm	54 (48.6)	113 (34.2)	0.007
**NHG**			
I	11 (9.9)	97 (29.5)	
II	45 (40.5)	133 (40.4)	
III	55 (49.5)	99 (30.1)	<0.001
**Node status**			
Negative	67 (65.7)	179 (61.3)	
Positive	35 (34.3)	113 (38.7)	0.43
Unknown	9	41	
**ER status**			
Negative	22 (20.0)	36 (10.9)	
Positive	88 (80.0)	294 (89.1)	0.01
Unknown	1	3	
**PR status**			
Negative	16 (14.8)	45 (13.8)	
Positive	92 (85.2)	281 (86.2)	0.79
Unknown	3	7	
n (%)	111 (25.0)	333 (75.0)	*p-value*
**Endocrine therapy (tamoxifen)**			
No	50 (55)	147 (59)	
Yes	41 (45)	103 (41)	
Unknown	20	83	*0.53*
**Chemotherapy**			
No	81 (89)	240 (95)	
Yes	10 (11)	12 (5)	
Unknown	20	81	*0.04*
**HER2 IHC**			
0	77 (73.3)	155 (48.9)	
1	13 (12.4)	89 (28.1)	
2	6 (5.7)	37 (11.7)	
3	9 (8.6)	36 (11.4)	*<0.001*
unknown	6	16	
**HER2 IHC**			
0 to 2	96 (91)	284 (89)	
3	9 (9)	36 (11)	*0.44*
Unknown	6	16	
**Ki67**			
0 to 10%	29 (27.6)	122 (39.5)	
11 to 25%	35 (33.3)	107 (34.6)	
>25%	39 (41.0)	80 (25.9)	*0.02*
Unknown	8	24	

ER = oestrogen receptor; HER2 = human epidermal growth factor receptor 2; IHC = immunohistochemistry; NHG = Nottingham histological grade; PR = progesterone receptor.

10% cut-off used for determination of hormone receptor status. Mann-Whitney test for comparison of medians and chi squared test for X × 2 tables. The categories marked as not done and unknown were not included in the analysis.

### HMG-CoAR is associated with an improved prognosis

**Figure 2:**
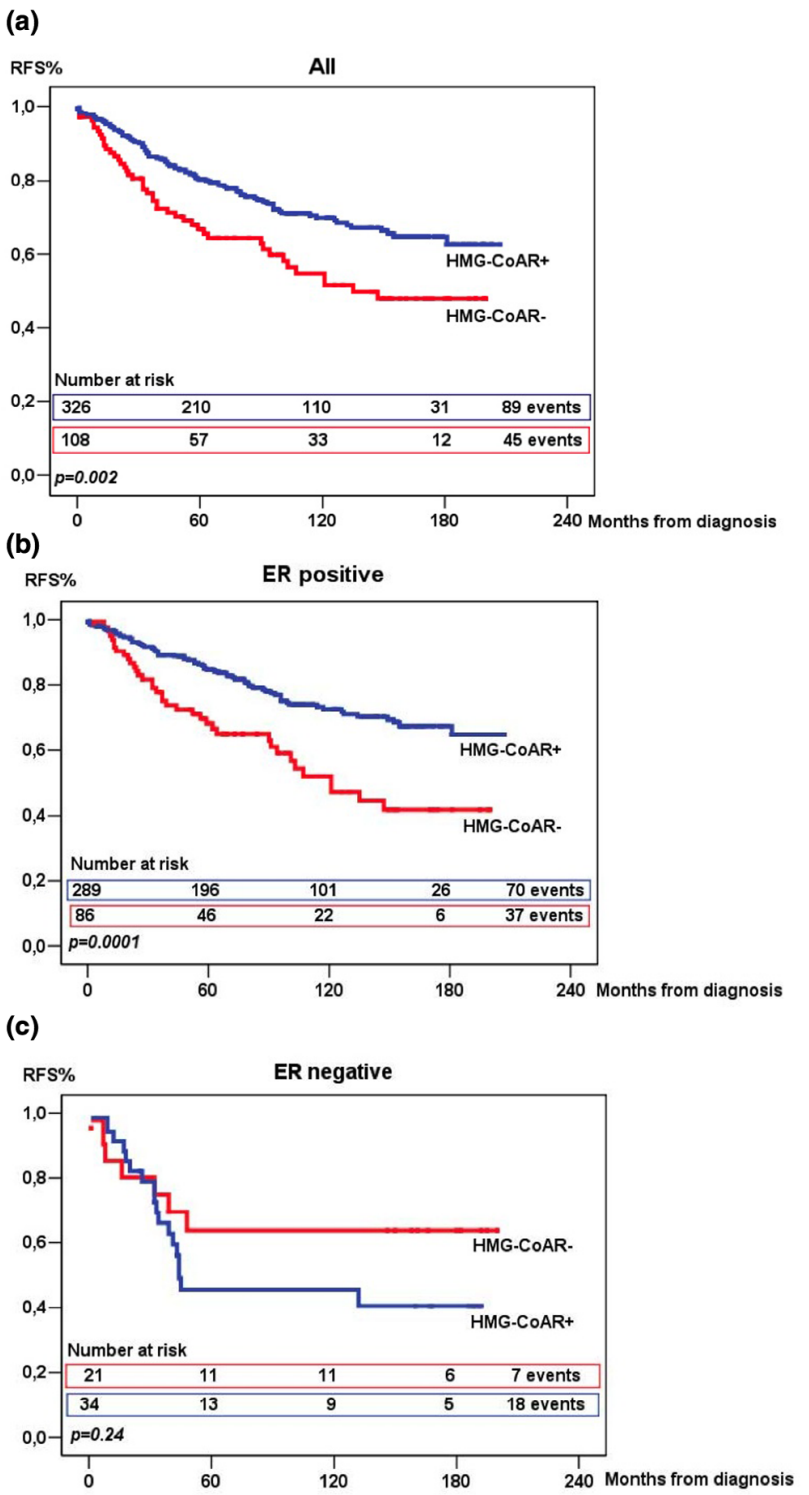
**Recurrence-free survival according to HMG-CoA reductase expression**. Kaplan-Meier estimates of recurrence-free survival according to HMG-CoA reductase expression, defined as negative (0) or positive (1 to 3), in all (a) patients, and in patients with (b) oestrogen receptor (ER) positive tumours and (c) ER-negative tumours.

Having demonstrated that HMG-CoAR protein expression was associated with a less aggressive phenotype, we proceeded to examine the relationship between HMG-CoAR expression and breast cancer recurrence. For survival analyses, a dichotomised variable defined as absent staining versus any staining was used. As illustrated in figure 2a, HMG-CoAR protein expression was associated with an improved RFS (p = 0.002). Subset analysis revealed that the favourable impact on outcome associated with HMG-CoAR expression was even more evident in ER-positive tumours (p < 0.0001), in contrast to the ER-negative subgroup, where the trend was reversed, but did not reach statistical signficance (p = 0.24). In the entire cohort, HMG-CoAR expression was not associated with an improved BCSS (p = 0.16); however, subset analysis again revealed an improved BCSS in ER-positive tumours (p = 0.05) and an inverse, but non-significant, trend for ER-negative tumours (p = 0.10) (data not shown). These data would suggest that HMG-CoAR expression is associated with a poor prognosis in ER-negative tumours. Stratification for PR and ER and/or PR positivity status provided similar results (data not shown).

We proceeded to perform a Cox regression proportional hazards analysis of RFS and BCSS, which demonstrated estimates of relative risks according to tumour-specific HMG-CoAR expression in univariate and multivariate analyses (Table 2). Multivariate analyses were adjusted for age at diagnosis, ER, tumour size, NHG, node status and HER2. Patients with HMG-CoAR-positive tumours had a significantly improved RFS compared with patients with HMG-CoAR-negative tumours independent of adjusted factors (relative risk [RR] = 0.64, 95% confidence interval [CI] 0.43 to 0.94, p = 0.002). When the analysis was confined to ER-positive patients (n = 375) the results became even more significant (RR = 0.47, 95% CI 0.30 to 0.73, p < 0.001), whereas no association between HMG-CoAR status and RFS was found in the cohort of ER-negative tumours (Table 2).

**Table 2:** Cox univariate and multivariate analysis of recurrence-free and breast cancer-specific survival according to HMGCoA-reductase expression in all, oestrogen receptor (ER) positive and ER-negative patients.

	**Recurrence-free survival**		**Breast cancer-specific survival**	
	
	**RR (95%CI)**	***p value***	**RR (95%CI)**	***p value***
	
**All patients**	***Univariate***		***Univariate***	
HMG-CoAR negative	1.00		1.00	
HMG-CoAR positive	0.57 (0.40 to 0.82)	0.002	0.64 (0.91 to 1.03)	0.07
	***Multivariate***		***Multivariate***	
HMG-CoAR negative	1.00		1.00	
HMG-CoAR positive	0.60 (0.40 to 0.92)	0.02	0.66 (0.39 to 1.11)	0.12
**ER positive**	***Univariate***		***Univariate***	
HMG-CoAR negative	1.00		1.00	
HMG-CoAR positive	0.46 (0.31 to 0.69)	<0.001	0.59 (0.34 to 1.00)	0.02
**ER negative**				
HMG-CoAR negative	1.00		1.00	
HMG-CoAR positive	1.68 (0.70 to 4.03)	0.25	2.47 (0.81 to 7.54)	0.11
Term of interaction*		0.004*		0.01*

**ER positive**	***Multivariate***		***Multivariate***	
HMG-CoAR negative	1.00		1.00	
HMG-CoAR positive	0.47 (0.30 to 0.73)	0.001	0.67 (0.38 to 1.17)	0.16
**ER negative**				
HMG-CoAR negative	1.00		1.00	
HMG-CoAR positive	1.45(0.35 to 5.97)	0.61	2.00 (0.37 to 10.93)	0.42
Term of interaction*		0.01*		0.11*

Multivariate analysis adjusted for Nottingham histological grade (I–II/III), age (continuous), nodal status(0/1), and tumour size (continuous). ER status (positive/negative, 10% cut-off), and human epidermal growth receptor 2 (HER2) immunohistochemistry 0 to 2 vs 3. * = Ŧ p value for term of interaction including ER (0/1), HMG-CoAR (0/1) and an interaction variable.

**Figure 3:**
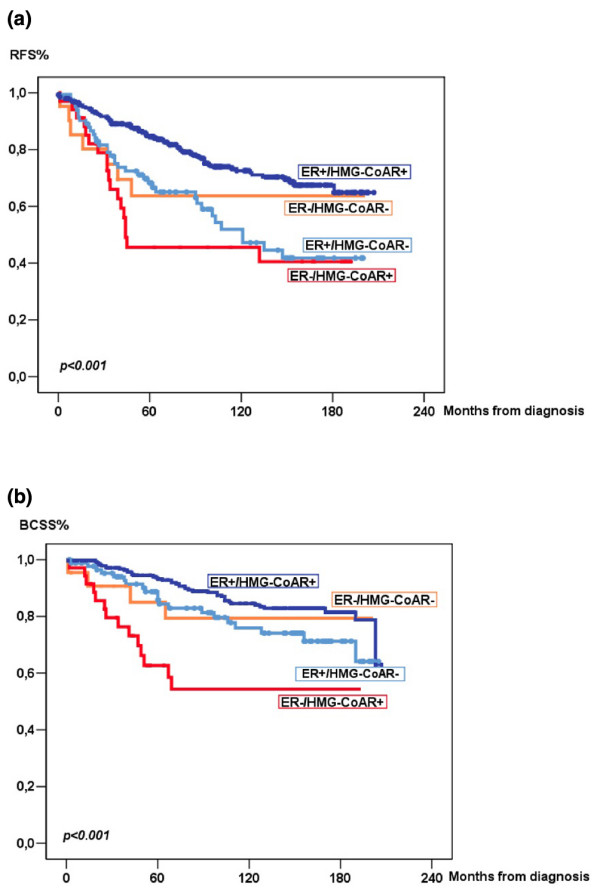
**Recurrence-free and breast cancer-specific survival according to combinations of oestrogen receptor (ER) status and HMG-CoA reductase expression**. Kaplan-Meier estimates of (a) recurrence-free and (b) breast cancer-specific survival according to combinations of ER-status (negative/positive) and HMG-CoA reductase expression (negative [0]/positive [1 to 3]).

In light of the apparent influence of ER status on the prognostic impact of HMG-CoAR expression, the relationship between ER and HMG-CoAR was examined in a Cox multivariate interaction analysis. This revealed a significant interaction between ER and HMG-CoAR expression related to both RFS (p = 0.004) and BCSS (p = 0.01) (table 2). When adjusted for established prognostic parameters, the interaction remained significant for RFS (p = 0.01) but not BCSS (p = 0.11). The different impact on survival in strata with different combinations of ER status and HMG-CoAR expression is further illustrated in figure 3, which identified the ER negative/HMG-CoAR positive group as those with the shortest survival, both in terms of RFS and BCSS.

Adjusting for adjuvant tamoxifen treatment did not alter the impact of HMG-CoAR on RFS or BCSS and the interaction between ER and HMG-CoAR was significant for RFS and BCSS in both univariate and multivariate analyses (data not shown).

### Correlations between HMG-CoAR and relevant clinicopathological parameters in ER-positive and ER-negative tumours

The apparent influence of HR status on the prognostic impact of HMG-CoAR led us to further examine the correlations between HMG-CoAR and established clinicopathological parameters stratified according to ER status (Table 3). This revealed an apparent discrepancy between the two groups, with a strongly positive association between HMG-CoAR positivity and nodal status in ER-negative patients (n = 58) (p < 0.001). Furthermore, no correlations between tumour size, NHG or proliferation and HMG-CoAR expression were evident in the ER-negative group. In contrast, in the ER-positive subgroup the association with favourable parameters such as small tumour size (p = 0.03), low NHG (p < 0.001) and Ki-67 negativity (p = 0.05) was retained. In addition, no association was seen between HMG-CoAR and nodal status in the ER-positive group.

**Table 3:** Correlation betweeen HMGCoAR expression and clinicopathological parameters in oestrogen receptor (ER) positive and ER-negative tumours.

	**ER-positive**			**ER-negative**		
	
**HMG-CoAR intensity**	0	1–3		0	1–3	
n (%)	88 (23)	294 (77)	*p value*	22 (38)	36 (62)	*p value*
**Age (years)**						
Median	66	65		57	59	
Range	28–89	35–96	0.43	27–81	34–90	0.38
≤50	15 (17)	45 (15)		7 (32)	8 (27)	
>50	73 (83)	249 (85)	0.69	15 (68)	28 (73)	0.42
**Tumour size**						
Median	20	15		22	17	
Range	1 to 100	1 to 100	<0.001	12 to 55	1 to 100	0.10
≥20 mm	48 (55)	147 (60)		9 (41)	19 (53)	
>20 mm	40 (45)	97 (40)	0.03	13 (59)	17 (47)	0.38
**NHG**						
I	11 (13)	95 (32)		0	1 (3)	
II	40 (45)	126 (43)		5 (23)	9 (25)	
III	37 (42)	72 (25)	<0.001	17 (77)	26 (72)	0.56
**Node status**						
Negative	48 (59)	167 (64)		19 (90)	11 (36)	
Positive	33 (41)	95 (36)	0.47	2 (10)	19 (63)	<0.001
Unknown	7	32		1	6	
						
**Tamoxifen**						
No	41 (55)	133 (60)		8 (50)	12 (44)	
Yes	33 (45)	87 (40)	0.45	8 (50)	15 (56)	0.73
Unknown	14	74		6	6	
						
**Chemotherapy**						
No	67 (91)	214 (96)		14 (88)	23 (85)	
Yes	7 (9)	8 (4)	0.05	2 (12)	4 (15)	0.84
Unknown	14	72		6	6	
						
**HER2 IHC**						
0	59 (71)	144 (51)		17 (81)	12 (34)	
1	13 (16)	85 (30)		0	5 (14)	
2	6 (7)	35 (12)		0	1 (3)	
3	5 (6)	19 (7)	0.02	4 (19)	17 (49)	0.007
Unknown	5	11		1	1	
						
**HER2 IHC**						
0 to 2	78 (94)	264 (93)		17 (80)	18 (51)	
3	5 (6)	19 (7)	0.82	4 (20)	17 (49)	0.02
Unknown	5	11		1	1	
**Ki67**						
0 to 10%	27 (32)	116 (42)		1 (5)	4 (12)	
11 to 25%	32 (38)	101 (37)		3 (15)	7 (21)	
>25%	25 (30)	58 (21)	0.05	16 (80)	23 (68)	0.30
Unknown	4	19		*2*	*1*	

ER = oestrogen receptor; HER2 = human epidermal growth factor receptor 2; IHC = immunohistochemistry; NHG = Nottingham histological grade.

10% cut-off used for determination of hormone receptor status. Mann-Whitney test for comparison of medians and chi-squared test for X × 2 tables. The categories marked as not done and unknown were not included in the analysis.

## Discussion

The present study confirms previous data identifying a correlation between tumour-specific HMG-CoAR expression and prognostic favourable clinicopathological parameters in breast cancer. In addition, the analysis of tumour specimens from a consecutive cohort of 498 breast cancer patients with long-term follow-up, revealed that HMG-CoAR expression is an independent predictor of RFS. Nevertheless, these data indicate that this beneficial influence is not extended to ER-negative tumours in which the impact on survival may in fact be the reverse. This assumption is supported by a significant interaction between ER status and HMG-CoAR expression in terms of both RFS and BCSS. The significant impact on prognosis was not only retained, but augmented when adjustment for adjuvant tamoxifen treatment was included in the multivariate analysis. Although this indicates that the prognostic value of HMG-CoAR is independent of tamoxifen treatment, we prefer not to draw any further conclusions, because this cohort, while providing robust prognostic data, was less well suited for evaluating treatment predictive effects. Such analyses should ideally be performed on tumour specimens from randomised trials. In this cohort, patients treated with tamoxifen had a significantly poorer survival compared with untreated subjects, reflecting the fact that they were diagnosed during an era where tamoxifen was primarily given to patients with more advanced disease. Also, as shown in table 3, a fraction of the ER-negative patients received tamoxifen, but this did not alter the effect of HMG-CoAR on survival in this subgroup.

The number of patients that had received adjuvant chemotherapy was too small (n = 23) to allow for evaluation of how this could have affected the results in the ER-negative group.

Despite a plethora of literature on the anti-neoplastic properties of statins, epidemiological data about their cancer preventing effect in general and breast cancer in particular are not conclusive [9,17-20]. Although breast cancer is a truly heterogeneous disease, consisting of several molecular subgroups that still need to be further refined in order to optimise treatment protocols, it is reasonable to assume that the mevalonate pathway plays a key role in certain subgroups. As shown in table 3, our data clearly show diverging associations to clinicopathological parameters in ER-positive and ER-negative tumours, with a strong association between HMG-CoAR expression and lymph node positivity in ER-negative tumours.

Previous studies have demonstrated that ER-negative cell lines are more sensitive to growth inhibition by statins than their ER-positive counterparts [10]. We are not aware of any studies related to statin response in ER-negative versus ER-positive tumours *in vivo*, but ongoing prospective trials will hopefully shed more light on this issue. ER-status alone, however, does not divide tumours into clinically relevant subgroups and additional surrogate markers are needed to select patients that would benefit from statin treatment. It is evident that the enzyme that is targeted by statins, HMG-CoAR, is expressed in breast cancer and that it, in the overall setting, predicts a good prognosis, but probably not in ER-negative tumours. These data suggest that an elevated HMG-CoAR expression may be a relevant surrogate marker of response to statin treatment, both in the adjuvant and chemopreventive setting, in ER-negative tumours.

Given the small number of ER-negative tumours present in this study and the risk of random associations with multiple subgroup analyses, this assumption must, however, be confirmed in prospective trials and in larger numbers of ER-negative tumours. Moreover, further studies are warranted to examine the role of HMG-CoAR as a predictor of statin treatment response in triple negative tumours (ER-negative, PR-negative and HER2-negative). Although HER2 amplification status was not available for the tumours in the present study, no firm conclusions can be drawn. Interestingly, in ER-negative and PR-negative tumours expressing HMG-CoAR (n = 40), a negative/low expression of HER2 (immunohistochemistry 0 to 2) was associated with a significantly shorter RFS (RR = 3.92, 95%CI 1.25 to 12.36, p = 0.02), but not BCSS (data not shown), compared with tumours with high expression of HER2 (immunohistochemistry 3). In contrast, among ER-negative/PR-negative/HMG-CoAR-negative tumours HER2 status had no prognostic impact on either RFS or BCSS (data not shown).

Data on statin use was not available for the patients in this cohort, but it can be assumed that the number of users was negligible during that period of time. In the Malmö Diet and Cancer Study, which was initiated in 1991, the number of statin users at baseline was too small, only 228 of 17,035 female participants, to allow for analyses of tumour-specific HMG-CoAR expression in relation to previous statin use [16]. Such studies would, however, be of interest, especially with regard to the reported lower incidence of ER-negative tumours among statin-users [10]. The focus of the current study was to investigate the impact of tumour-specific HMG-CoA reductase expression on disease outcome in primary breast cancer with possible implications to its relevance in a tumour biological, not epidemiological, context. However, given the association between tumour-specific HMG-CoAR expression and ER-positive tumours, which we have now observed in two large breast cancer cohorts, it would be interesting to see whether this association is altered in ER-positive tumours in previous statin users. It can not readily be hypothesised that the use of statins, which inhibit HMG-CoAR expression, would promote the incidence of a tumour phenotype linked to a higher expression of the enzyme. It would also be of interest to examine whether HMG-CoAR expression is affected in ER-negative tumours that still occur among statin users, that is, if the proportion of HMG-CoAR expressing tumours is reduced.

## Conclusion

The target enzyme for cholesterol-lowering statins, HMG-CoAR, is associated with improved prognosis among ER-positive breast cancer patients, whereas ER-negative patients seem to have a better outcome when HMG-CoAR is absent. Future randomised trials are warranted to clarify the potential beneficial effects of statins in the adjuvant and metastatic setting.

## Abbreviations

BCSS: breast cancer specific survival; CI: confidence interval; ER: oestrogen receptor; HER2: human epidermal growth factor receptor 2; HMG-CoAR: 3-hydroxy-3-methylglutharyl-coenzyme A reductase; HR: hormone receptor; MDCS: Malmö Diet and Cancer Study; NHG: Nottingham histological grade; PR: progesterone receptor; RFS: recurrence free survival; RR: relative risk; TMA: tissue microarray.

## Competing interests

The authors declare that they have no competing interests.

## Authors' contributions

SB performed immunohistochemical assessment, statistical analysis and drafted the manuscript. AJ participated in the design of the study, data interpretation and draft of the manuscript. LR assisted with the statistical analysis and revision of the manuscript. FP participated in the conception and design of the study. DB performed immunohistochemical assessment, statistical analysis and drafted the manuscript. KJ participated in the conception and design of the study, statistical analysis, and helped to draft and revise the manuscript.
